# High Doses of Caffeine Increase Muscle Strength and Calcium Release in the Plasma of Recreationally Trained Men

**DOI:** 10.3390/nu14224921

**Published:** 2022-11-21

**Authors:** Luis H. B. Ferreira, Scott C. Forbes, Marcelo P. Barros, André C. Smolarek, Alysson Enes, Antonio H. Lancha-Junior, Gabriel L. Martins, Tacito P. Souza-Junior

**Affiliations:** 1 Metabolism, Nutrition and Strength Training Research Group (GPMENUTF), Federal University of Paraná (UFPR), Curitiba 81531-980, PR, Brazil; 2 Department of Physical Education Studies, Brandon University, Brandon, MB R7A 6A9, Canada; 3 Institute of Physical Activity Sciences and Sports, Universidade Cruzeiro do Sul, São Paulo 07115-000, SP, Brazil; 4 Laboratory of Clinical Investigation: Experimental Surgery (LIM 26), Clinic’s Hospital of Medical School, University of Sao Paulo, Sao Paulo 05508-030, SP, Brazil

**Keywords:** ergogenic, one repetition maximum, athletic performance, xanthine, sarcoplasmic reticulum

## Abstract

The effects of acute caffeine supplementation on muscular strength remain unclear. We examined the effects of two different doses of caffeine on muscle strength and calcium in plasma compared to placebo using a crossover, randomized, double-blind, placebo-controlled design. Twenty-one (*n* = 21) recreationally resistance-trained participants were randomly assigned into three experimental conditions: 6 mg·kg bw^−1^ of caffeine (CF6); 8 mg·kg bw^−1^ of caffeine (CF8); or placebo (PLA), with a 7-day washout period between conditions. Muscular strength assessments were made for both upper (bench press) and lower body muscles (squat and deadlift). Calcium release in plasma was measured on five different occasions. Bench press (CF8: 100.1 ± 1.9 kg; PLA: 94.2 ± 2.5 kg), deadlift (CF8: 132.8 ± 3.5 kg; PLA: 120.7 ± 5.7 kg), and squat (CF8: 130.1 ± 4.9 kg; PLA 119.4 ± 5.4 kg) strength were all significantly (*p* < 0.001) improved in CF8 compared to PLA. Calcium release in plasma was significantly increased in CF8, whereas no changes were observed in CF6 or PLA. Overall, 8 mg·kg bw^−1^ of caffeine appears to be an effective dose to optimize upper and lower body muscular strength and calcium release in recreationally trained participants.

## 1. Introduction

Caffeine is an alkaloid derived from methylxanthines (1,3,7-trimethylxanthine) and is one of the most consumed psychoactive substances worldwide [1]. Caffeine is renowned as an effective ergogenic aid to increase endurance performance [2]. Mechanistically, caffeine acts as an adenosine antagonist to reduce the perception of pain and exertion during exercise [3], as well as to improve muscle relaxation time by optimizing calcium mobilization in the sarcoplasmic reticulum and by altering the sodium–potassium ATPase pump activity. In terms of energy metabolism, caffeine is a powerful inhibitor of cyclic adenosine-monophosphate (cAMP) phosphodiesterase in the liver and other tissues, which is purported to sustain glycogenolysis in skeletal muscle and hyperglycemia during exercise. There is little evidence to support the hypothesis that caffeine has ergogenic effects as a result of enhanced fat oxidation [4].

Despite the well-known benefits of caffeine to improve endurance performance, there is limited research as to its effects on muscular strength [5]. Caffeine may enhance muscular strength by altering the capacity of calcium (Ca^2+^) release through the sensitization of Ca^2+^ channels, inducing Ca^2+^ discharge from the sarcoplasmic reticulum (SR) [6]. Moreover, it has been proposed that the onset of fatigue is directly associated with the combination of decreased Ca^2+^ release from the SR and reductions in myofibril Ca^2+^ sensitivity [7,8,9]. Caffeine supplementation is associated with improvements in Ca^2+^ release in a dose-dependent manner, whereby only doses higher than 8 mg kg∙bw^−1^ seem to effectively increase both the release and the sensitivity to Ca^2+^ [6,10], fostering improvements in muscular strength [11].

Therefore, although the efficacy of caffeine on endurance performance is well-established [1,2,12,13], there are conflicting results regarding its effectiveness on muscular strength [1,5,13,14,15,16]. Some results show no effect of 6 mg kg∙bw^−1^ of caffeine on 1-RM bench press or leg press strength compared to placebo [17], while other results found improvements in upper body strength with the same dose of caffeine (6 mg∙kg bw^−1^) [18]. A recent meta-analysis revealed that caffeine enhances 1-RM muscular strength compared to placebo, but interestingly the sub-analysis found no effect on lower body strength [19]. These authors concluded that “given the relatively small number of studies, future research is warranted” [19]. 

Given that Ca^2+^ release apparently follows a dose response pattern related to caffeine, the aim of the present study was to analyze the effects of different doses of caffeine on muscular strength and on Ca^2+^ release in the plasma of recreationally trained men. We hypothesized that higher doses of caffeine (8 mg∙kg bw^−1^) would significantly improve muscular strength, which would be accompanied by higher Ca^2+^ release in plasma compared to lower doses (6 mg∙kg bw^−1^). In addition, we hypothesized that both doses of caffeine would induce more substantial changes than placebo (PLA).

## 2. Materials and Methods

### 2.1. Participants 

Twenty-one recreationally active males (age: 19.6 ± 0.8 years; height: 173.8 ± 5.5 cm; body mass: 77.1 ± 8.2 kg; body fat: 11.8 ± 3.2%; strength training adherence: 5.1 ± 1.3 h/wk) volunteered to participate in this study. To reduce the potential for bias, the study employed a double-blind, randomized, crossover design. All participants met the following inclusion criteria: (1) actively engaged in at least four h∙wk^−1^ of strength training for the 12 months prior to the beginning of the study; (2) absence of any pathology or injury that would alter muscle biology and performance; (3) be a non-smoker; and (4) have a habitual daily intake of less than 150 mg∙d^−1^ of caffeine [17], which was analyzed with a self-reported questionnaire. An a priori power analysis for repeated-measures Analysis of Variance (ANOVA) for within-between interaction with an expected effect size (ES) f of 0.35, alpha of 0.05, r between repeated measures of 0.80, and statistical power of 80% indicated that the required sample size was *n* = 18. Power analysis was performed using G*Power (version 3.1; Dusseldorf, Germany). We further estimated that ~15% of the volunteers would not complete the whole study protocol and, as such, three additional participants were recruited to ensure a sufficient sample. All participants signed an informed consent. The study was approved in the UFPR institutional review board under the protocol CAAE: 58116816.4.0000.0102 and accepted at the Brazilian Clinical Trials Registry (RBR-52twgg), adhering to CONSORT guidelines.

### 2.2. Familiarization Trial and Repetition Maximum Assessment 

All participants were instructed to arrive at the laboratory wearing active wear and to refrain from any food or beverage containing caffeine and intense exercise within 48 h prior to the experimental sessions. During their first visit to the laboratory, anthropometric parameters were assessed, including body mass and body fat percentage (%BF), measured with a tetra-polar bioelectrical impedance analyzer (Model TFB-310 Tanita^®^; Tokyo, Japan), and standing height, measured with a stadiometer (Holtain Harpen ^®^; Crymych, Dyfed, UK) fixed to a wall [20]. Following anthropometric assessments, participants performed a warm-up session using a load of approximately 40% of one-repetition maximum (1-RM) for 20 repetitions in the squat, bench press, and deadlift. Subsequently, participants received instructions regarding the 10-repetition maximum (10-RM) test, selecting enough weight to perform between 1 and 10 repetitions in each one of the three exercises. At the end of each test and 30 min after the testing session, participants provided their rating of perceived exertion (RPE) using the OMNI scale [21]. A food intake questionnaire was employed to monitor and control the diet of participants between the experimental days [22].

### 2.3. Strength Test and 10-RM Protocol

Three exercises were selected to assess the dose–response relationship between caffeine supplementation and strength performance. Each participant was instructed to perform three attempts to determine their 10-RM for each exercise selected (bench press, squat, and deadlift). Individual attempts and tests were separated by four minutes of passive rest. The attempt with the highest amount of weight and repetitions executed with proper form was recorded (18). The results were used to calculate the 1-RM for all exercises (7), providing an estimation of the strength levels from the sample under different conditions (i.e., placebo/6/8 mg∙kg bw^−1^). 

### 2.4. Supplementation and Biochemical Analyzes Protocol

**Figure 1:**
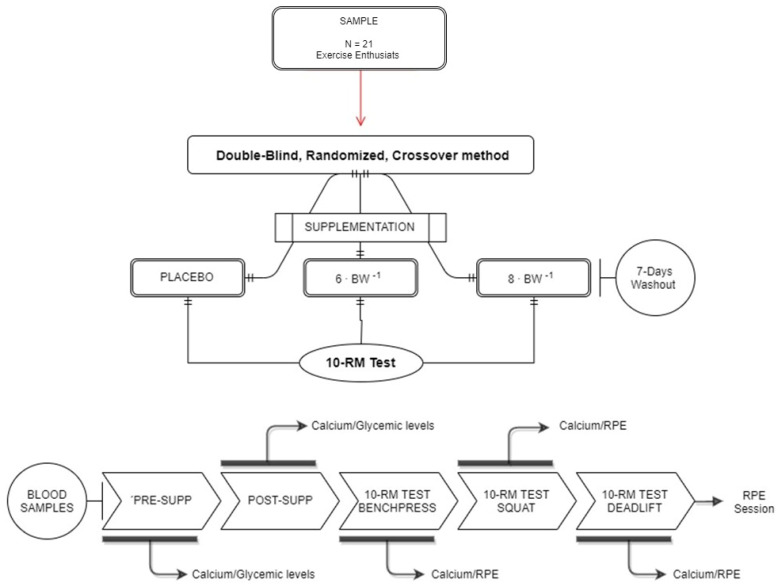
Study Design.

After the initial familiarization session, participants were instructed to arrive at the lab facilities on three different occasions in a fasted state aiming to test the proposed conditions in the study. Upon arrival at the laboratory, participants received an isocaloric shake containing 40 g of maltodextrin and 40 g of dextrose, totaling 480 kcal. Approximately 30 min after the meal, participants randomly received either 6 mg∙kg bw^−1^ (CF6) or 8 mg∙kg bw^−1^ (CF8) of caffeine or the placebo (PLA). All the procedures of caffeine/placebo intake were conducted using capsules of similar appearance and weight. In the placebo capsules, we used inert substances to provide the capsules’ weight. Each experimental condition was separated by a one-week washout [14]. The glycemic levels and Ca^2+^ concentrations were measured in plasma using an Abbott c16000 Clinical Chemistry Analyzer (Abbott Diagnostics Inc, Lake Forest, IL, USA). A blood sample was obtained from the antecubital veins at five different time-points per session: (i) before supplementation; (ii) 45 min after supplementation; (iii) immediately after the bench press test; (iv) immediately after the squat test; and (v) immediately after the deadlift test. The experimental design is presented in (Figure 1). 

### 2.5. Statistical Analysis 

All outcome measures were reported as mean ± SD and were assessed using SPSS Version 25.0 (SPSS™, Inc., Chicago, IL, USA). The Shapiro–Wilk test assessed the normality of the data distribution. A one-way ANOVA (mixed model) with repeated measurements was applied to compare differences between muscle strength, whereas a two-way ANOVA with repeated measures was used to analyze Ca^2+^ release and RPE with different doses of caffeine and placebo. For all dependent variables, Tukey’s post-hoc test was performed to identify differences between means when a significant F ratio was obtained. Cohen’s d effect sizes (ESs) were reported for each outcome and interpreted using the classification scale proposed by Rhea (2004) [23], where values < 0.41 represent a small ES, 0.41–0.70 a moderate ES, and >0.70 a large ES. Statistical significance was established a priori at *p* < 0.05.

## 3. Results

No differences were observed in total calories ingested the day prior to each experimental condition (see Table 1). Following each testing condition (CF6, CF8, and PLA), the participants were asked to predict which supplement condition (low or high caffeine, placebo) they had consumed. Among the participants, 37.5% correctly identified their consumption of the lower caffeine dose, whereas 43.7% correctly identified consumption of the higher dose condition. There were no self-reported cases of digestive discomfort or any other side effects in any of the conditions. 

### 3.1. Strength Analysis 

#### 3.1.1. 1-RM on Bench Press

**Figure 2:**
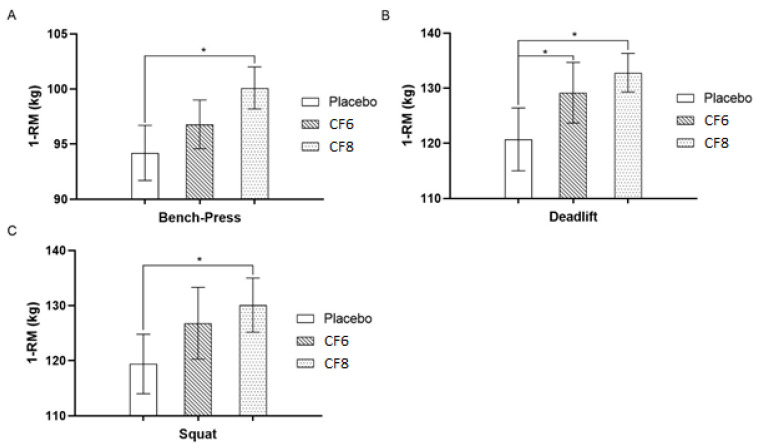
Results of 1-RM on Bench Press, Deadlift and Squats. Figure 1. (**A**) Effect of different doses of caffeine during the bench press strength analysis; (**B**) Effect of different doses of caffeine during the deadlift strength analysis; (**C**) Effect of different doses of caffeine during the squat strength analysis. * = significant difference (*p* < 0.05) compared to placebo.

Bench press strength results (with post hoc analyses) revealed a statistically significant effect and a relatively large associated ES with high doses of caffeine supplementation (CF8 versus PLA; *p* = 0.01; Figure 2A; CF8: 101.1 ± 1.9 kg; and PLA: 94.2 ± 2.5 kg; *p* = 0.01; ES = 0.79). No other statistically significant differences were observed between conditions (*p* > 0.05). However, a moderate ES was observed between CF8 and CF6 (0.69) and between CF6 and placebo (0.48). 

#### 3.1.2. 1-RM on Deadlift

Deadlift strength scores were similar to those observed for bench press (*p* = 0.001) (Figure 2B), with a statistically significant difference and a relatively large associated ES demonstrated between CF8 and placebo (132.8 ± 3.5 kg to 120.7 ± 5.7 kg, with *p* = 0.03; ES = 0.78). A small ES (0.36) was observed between CF8 and CF6, whereas a moderate ES (0.60) was observed between CF6 and placebo. 

#### 3.1.3. 1-RM on Squat

In agreement with previous strength results, squat strength results revealed a statistically significant main effect (*p* = 0.01) (Figure 2C). Post hoc analysis showed a statistically significant difference between CF8 and placebo, with a relatively large associated ES (130.1 ± 4.9 kg to 119.4 ± 5.4 kg, with *p* = 0.01; ES = 0.72). No other statistical differences were observed between conditions (*p* > 0.05), although a small ES (0.27) was observed between CF8 and CF6 and a moderate ES (0.52) between CF6 and placebo. 

### 3.2. Calcium Release in Plasma 

**Figure 3:**
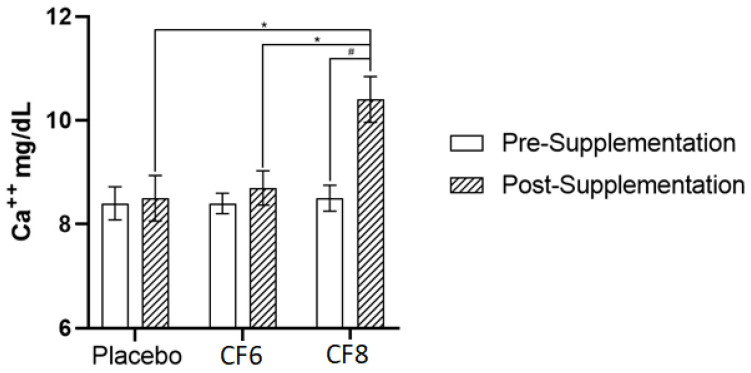
Calcium release 45 min after supplementation. * = significant differences (*p* < 0.05) compared to placebo and CF6; # = significant difference (*p* < 0.05) compared to pre-supplementation.

**Figure 4:**
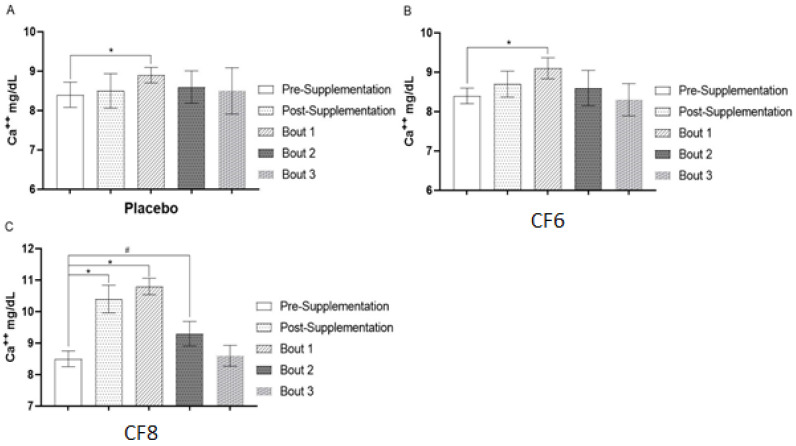
Calcium behavior after supplementation. Figure 2. (**A**) Calcium behavior from pre-supplementation with placebo until the last strength bout analyzed; (**B**) Calcium behavior from pre-supplementation with lower doses of caffeine until the last strength bout analyzed; (**C**) Calcium behavior from pre-supplementation with higher doses of caffeine until the last strength bout analyzed. * = significant differences (*p* < 0.05) compared to pre-supplementation; # = significant difference (*p* < 0.05) between Bout 2 vs. pre-supplementation.

There was a statistically significant between-condition interaction for calcium release in plasma (*p* = 0.01). Pre-/post-caffeine supplementation did not affect Ca^2+^ release in plasma of the CF6 condition compared to placebo (*p* > 0.05). However, CF8 showed a significant increase in plasma Ca^2+^ content 45 min after supplementation: pre 8.5 ± 0.3 mg/dL; post 10.4 ± 0.44 mg/dL; *p* = 0.001) (Figure 3). Moreover, post CF8 showed a higher Ca^2+^ release in plasma than both placebo (10.4 ± 0.44 mg/dL to 8.5 ± 0.4 mg/dL; p = 0.001; ES = 0.95) and CF6 (10.4 ± 0.44 to 8.7 ± 0.36; p = 0.001; ES = 0.89). Furthermore, placebo, CF6, and CF8 displayed a higher Ca^2+^ release in plasma after the first exercise bout (*p* < 0.001), as shown in Figure 4. Ca^2+^ release was only significantly higher after the second bout in CF8, compared to their respective pre-test values (8.4 ± 0.32 mg/dL to 9.4 ± 0.41 mg/dL; *p* < 0.001). No other statistically significant differences were observed (*p* > 0.05). 

### 3.3. Rating of Perceived Exertion (RPE)

Results of the RPE showed that CF8 volunteers perceived higher efforts compared to placebo: 7.37 ± 1.14 to 4.87 ± 1.36, respectively (*p* = 0.04). No other statistically significant differences were observed among RPE scores (CF6: 7.37 ± 1.14; CF8: 6.33 ± 1.24).

## 4. Discussion

Controversy persists as to the ergogenic effects of caffeine supplementation on muscular strength with some studies showing no benefit [13,16,17,24] and others demonstrating positive effects [25,26,27,28]. However, it is conceivable that the reason for the conflicting results may be associated with the ingested dose of caffeine. The aim of the present study was to examine the effects of two different doses of caffeine on maximal strength, Ca^2+^ release in plasma, and fatigability (here assessed via RPE). The current literature is somewhat equivocal regarding the relationship between caffeine intake and strength. To the best of our knowledge, this is the first study comparing the effects of moderate and high doses of caffeine (6 and 8 mg∙kg bw^−1^, respectively) on the strength of upper and lower body muscles in recreationally trained participants. We found that a moderate dose of caffeine (6 mg∙kg bw^−1^) did not significantly improve strength output, although a moderate ES was observed for bench press, deadlift, and squat strength compared to placebo. A higher dose of caffeine (8 mg∙kg bw^−1^) however, did show significant strength-related benefits compared to placebo.

The current data contradict some previous studies [13,16,17] that did not find an effect of caffeine on strength performance. Apart from the different methodologies, one possible reason for the divergent findings might be related to the lower doses used in those studies (≤6 mg∙kg^−1^) compared to those employed herein (6 to 8 mg∙kg^−1^). Two other studies using 6 mg∙kg^−1^ doses [25,27] demonstrated a significant strength improvement from caffeine supplementation, which conflicts with our findings for CF6. A possible explanation for these discrepancies may be related to the specificity of the strength training applied and the physical activity level of the participants. Both of the aforementioned studies that showed ergogenic effects on strength using lower doses of caffeine used resistance-trained participants with more than two years of training experience, while the participants of the present study were only required to have more than 12 months. The longer period of exposure to resistance training may have resulted in the development of a more sensitive voluntary neuromuscular activation [8], which in turn could have enhanced the ability of the participants to improve strength with lower caffeine dosages [29]. However, the reasons for the discrepancies observed are not completely clear considering that different methodologies were employed during those studies, suggesting that more investigations must be performed to fully understand these effects. 

The ergogenic effect of caffeine on strength is also dependent on the dose of the stimulant relative to the normal caffeine consumption by the participants (chronic exposure) [19]. Concerning chronic exposure, some authors recommend that a caffeine dosage of 3–9 mg·kg^−1^ is both safe and ergogenic for various exercise-related outcomes [30,31,32]. However, it is not common to use doses higher than 6 mg·kg^−1^, with a lack of studies investigating the impact of higher doses on the strength performance of participants with high, habitual caffeine consumption [33,34]. The current literature has only investigated the effects of different amounts of caffeine in chronic caffeine consumers on aerobic exercise performance, showing inconclusive results [35,36]. The present study recruited participants with a low habitual caffeine intake (≤150 mg caffeine/day), which in theory may have enhanced the sensitization of participants towards the stimulant. As such, our findings should not be generalized to individuals that habitually ingest a large amount of caffeine.

To the best of our knowledge, only one study [26] investigated the effects of similar doses of caffeine (8 mg·kg bw^−1^) in recreationally trained individuals with a low habitual caffeine consumption (approximately 172 mg per week). Results indicated that caffeine supplementation improved the activation of maximal voluntary torque (MVT) in the quadriceps muscles [26]. These findings support the results presented here since CF8 showed ergogenic effects on muscle strength in the bench press, deadlift, and squat compared to placebo (Figure 2). This enhancement of muscle strength may be related to increases in muscle fiber recruitment and muscle power induced by higher dosages of caffeine, which, at least in part, may be due to an increased Ca^2+^ release in the sarcoplasmic reticulum combined with enhanced Ca^2+^ sensitivity in muscle cells [37]. Unfortunately, we did not shed light on the proper indexes of energy metabolism in muscles or plasma to evaluate the effectiveness of caffeine on cAMP phosphodiesterase inhibition, hepatic/muscle glycogenolysis, or glycemia. Moreover, caffeine or its metabolites could also chelate harmful “free” iron ions in plasma and tissues, which could limit the accessibility of these redox-active catalysts that promote the overproduction of reactive oxygen and nitrogen species (ROS/RNS) during exercise [38,39]. All these mechanisms are worthy of further investigation.

**Figure 5:**
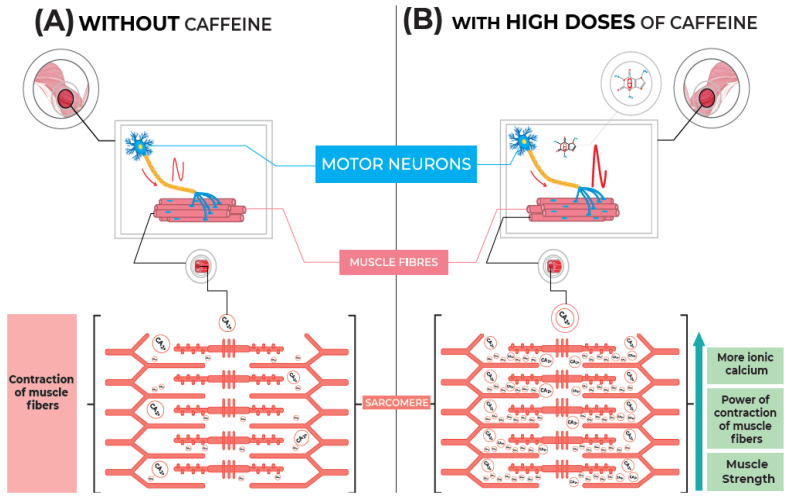
Possible mechanism of action of caffeine in increasing muscle strength at high doses. Figure 5. (**A**) Representation of the neuromuscular stimulus on the release of ionic calcium in muscle fibers and its impact on the increase in the power of contraction triggered by actin and myosin filaments in a “basal” condition (or on the placebo condition); (**B**) Representation of the more pronounced neuromuscular stimulus after the administration of high doses of caffeine, providing a more pronounced increase in the release of calcium ions and, probably, increasing the power of contraction of muscle fibers and muscle strength.

Calcium dynamics in muscle and plasma is another possible mechanism whereby caffeine supplementation could improve muscle strength. Previous research demonstrated that a high dose of caffeine (≤6 mg·kg^−1^) was able to promote enhancement of Ca^2+^ release compared with lower doses and placebo [40,41]. This is in accordance with the hypothesis that higher doses are needed to observe alterations in calcium-dependent pathways [42]. Importantly, muscle contraction speed is critically dependent on Ca^2+^ concentrations, and our findings are consistent with previous research with the highest caffeine dose (CF8) promoting an increase in Ca^2+^ release in plasma [43]. In theory, the higher caffeine dose may have enhanced strength performance due to an increased muscle contractility associated with Ca^2+^ release. In our study, the CF8 condition significantly increased Ca^2+^ release after the supplementation period when compared to the other experimental conditions (Figure 3). Furthermore, Ca^2+^ concentration in plasma remained elevated during the entire test session (Figure 4). Thus, we speculate that the initial increase in Ca^2+^ release, prior to the exercises, could have altered the neuromuscular action potential necessary for the strength enhancement in CF8. We illustrate this possible mechanism in Figure 5. Future studies investigating these mechanisms using higher caffeine dosages (>6mg/ bw^−1^) is warranted.

Previous research shows a relationship between caffeine ingestion and reductions in RPE during exercise. In the present study, we found that the higher dose of caffeine (CF8) resulted in a significantly larger RPE score than CF6 or placebo. Despite these findings, strength performance was significantly higher in CF8, indicating that the increased RPE did not negatively affect performance.

The present study has some limitations that should be considered when interpreting the use of caffeine as a potential ergogenic aid. First, the findings are specific to anhydrous caffeine; thus, these results cannot necessarily be generalized to the effect of caffeine-containing drinks or other substances that involve a combination of other ingredients. In addition, the sample was comprised of only recreationally trained young men, therefore the findings cannot necessarily be generalized to other populations, including women, youth, and older adults, as well as inactive or well-trained individuals. 

## 5. Conclusions

In conclusion, the present study revealed that a higher dose of caffeine may help to optimize strength performance. An enhanced Ca^2+^ release in plasma, even after the first bout of exercise, was only observed when higher doses of caffeine were applied and during two of three exercise bouts afterwards. These results suggest a relationship between Ca^2+^ release in plasma and strength-mediated effects of caffeine consumption in a dose-dependent fashion. Although higher doses of caffeine are related to improvements in strength, higher levels of RPE were also observed.

## Figures and Tables

**Table 1:** Calorie consumption 1 day before trials.

	1-Day Before Day 1	1-Day Before Day 2	1-Day Before Day 3
kcal	2587 ± 430	2375 ± 441	2690 ± 355
Carbohydrates (kcal)	1241 ± 208	1235 ± 229	1479 ± 195
Protein (kcal)	905 ± 151	902 ± 167	807 ± 106
Fats (kcal)	439 ± 71	238 ± 45	404 ± 54

kcal: kilocalories.

## Data Availability

The data presented in this study are available on request from the corresponding author (LHBF) with an appropriate reason.
